# The COVID-19 pandemic and kangaroo mother care: What should we do?

**DOI:** 10.18332/ejm/121095

**Published:** 2020-05-12

**Authors:** Sevil Hakimi

**Affiliations:** 1 Department of Midwifery, Tabriz University of Medical Science, Tabriz, Iran

**Keywords:** kangaroo mother care, COVID-19

Kangaroo mother care (KMC) is a model of care that is an alternative to the incubator for preterm newborns. The World Health Organization (WHO) has recommended this type of care in both developed and developing countries as soon as the premature neonate is clinically stabilized^1^.

The COVID-19 pandemic originated in the Hubei province of China in December 2019^2^. The virus spread rapidly and by March 2020 over 100 countries were affected^3^. Owing to the high contagion and fatality rate of the virus and the WHO declaration of COVID-19 as a pandemic, routine medical care was impacted and consequently the rate of KMC may have also suffered.

Clinical evidence shows KMC could be effective in improving the newborn's neurodevelopment outcomes, stabilize preterm newborn's physiological function and decrease maternal distress following the birth. KMC is effective in the initiation of exclusive breastfeeding^4^. Human milk is a unique dynamic nutrition source for the newborn during the first 6 months of life. Human milk directly contributes to innate immunity of the newborn by shaping gut microbiota and milk oligosaccharides^5^.

Recently, the WHO recommended that mothers and newborns should not be separated. The dyads should enable the practice of KMC even in cases of suspected or confirmed COVID-19 by using personal protective equipment and the disinfection of used surfaces^6^.

We urge clinicians, midwives and policy makers to keep neonatal care at the frontline^7^, and as such consider KMC in the neonatal wards, with the use of all related precautions.
